# HIF-1α regulates cellular metabolism, and Imatinib resistance by targeting phosphogluconate dehydrogenase in gastrointestinal stromal tumors

**DOI:** 10.1038/s41419-020-02768-4

**Published:** 2020-07-27

**Authors:** Kangjing Xu, Zhongyuan He, Ming Chen, Nuofan Wang, Diancai Zhang, Li Yang, Zekuan Xu, Hao Xu

**Affiliations:** 1 Department of General Surgery, The First Affiliated Hospital of Nanjing Medical University, Nanjing, 210029 China; 2 Jiangsu Key Lab of Cancer Biomarkers, Prevention and Treatment, Jiangsu Collaborative Innovation Center for Cancer Personalized Medical University, Nanjing, 211166 China

**Keywords:** Sarcoma, Gastrointestinal diseases

## Abstract

The pentose phosphate pathway (PPP) plays a critical role in maintaining cellular redox homeostasis in tumor cells and macromolecule biosynthesis. Upregulation of the PPP has been shown in several types of tumor. However, how the PPP is regulated to confer selective growth advantages on drug resistant tumor cells is not well understood. Here we show a metabolic shift from tricarboxylic acid cycle (TCA) to PPP after a long period induction of Imatinib (IM). One of the rate-limiting enzymes of the PPP-phosphogluconate dehydrogenase (PGD), is dramatically upregulated in gastrointestinal stromal tumors (GISTs) and GIST cell lines resistant to Imatinib (IM) compared with sensitive controls. Functional studies revealed that the overexpression of PGD in resistant GIST cell lines promoted cell proliferation and suppressed cell apoptosis. Mechanistic analyses suggested that the protein level of hypoxia inducible factor-1α (HIF-1α) increased during long time stimulation of reactive oxygen species (ROS) produced by IM. Importantly, we further demonstrated that HIF-1α also had positive correlation with PGD, resulting in the change of metabolic pathway, and ultimately causing drug resistance in GIST. Our findings show that long term use of IM alters the metabolic phenotype of GIST through ROS and HIF-1α, and this may contribute to IM resistance. Our work offers preclinical proof of metabolic target as an effective strategy for the treatment of drug resistance in GIST.

## Introduction

Gastrointestinal stromal tumor (GIST) is the most common sarcoma^1^, often harboring gain-of-function mutations in the KIT receptor tyrosine kinase^2^. While IM has revolutionized the treatment of GIST through targeting KIT^3^, resistance and disease progression often develop within 2 years^4^. The emergence of secondary mutations, activation of alternative survival pathways, changes in transporters or enzymes, pharmacokinetic metabolic variability and unknown effects of KIT or PDGFR polymorphisms, all have been considered as possible causes of drug resistance^5,6^. Promising second-line and third-line therapies have only provided minimal benefit, thus highlighting the need for clarifying the molecular mechanism of resistance to IM and improving the long-term prognosis of patients by discovering novel therapeutic approaches^7,8^.

Abnormal metabolism is a significant characteristic of tumors. The metabolic flux in tumor cells is markedly reprogrammed to provide elevated amounts of building blocks for rapid cell growth and metastasis^9,10^. However, it is not well understood how tumor cells reset the metabolic phenotype to promote tumor cell survival and cell proliferation. The PPP plays a vital role in meeting the cellular demands for anabolic biosynthesis and providing anti-oxidative defense^11^. Its generation including ribose-5phosphate and NAPDH, can be used in de novo synthesis of RNA and DNA to promote cell survival and be an important antioxidant to diminish high levels of ROS generated during rapid cell proliferation, respectively. Knock-down of key enzymes in the PPP inhibits tumor growth and sensitizes tumor cells to oxidative stress^12,13^. PGD is considered as the pacesetter of the PPP. However the increase of PGD in PPP leading to IM resistance has not been examined.

ROS are formed as a natural byproduct of the normal metabolism of oxygen and have important roles in cell signaling and homeostasis^14–16^. Excessive ROS production results in cell cycle arrest and apoptosis in tumor^17–19^. We supposed ROS also played an important role in GIST suppressed by IM.

The effects of IM on GIST glycometabolism are largely unknown. Because IM treatment dramatically promotes tumor cell quiescence and usually leads to drug resistance in GIST, we hypothesized that metabolism alterations contribute to IM resistance^20^. Herein, we investigate the role of PPP in GIST and show that PGD overexpression in PPP plays an important role in eliminating ROS. Furthermore, we found repressing HIF-1α in resistant GIST cells could elevate the production of ROS, sensitizing GIST cells to IM.

## Results

### IM-resistant cells have upregulated glycometabolism level

**Fig. 1:**
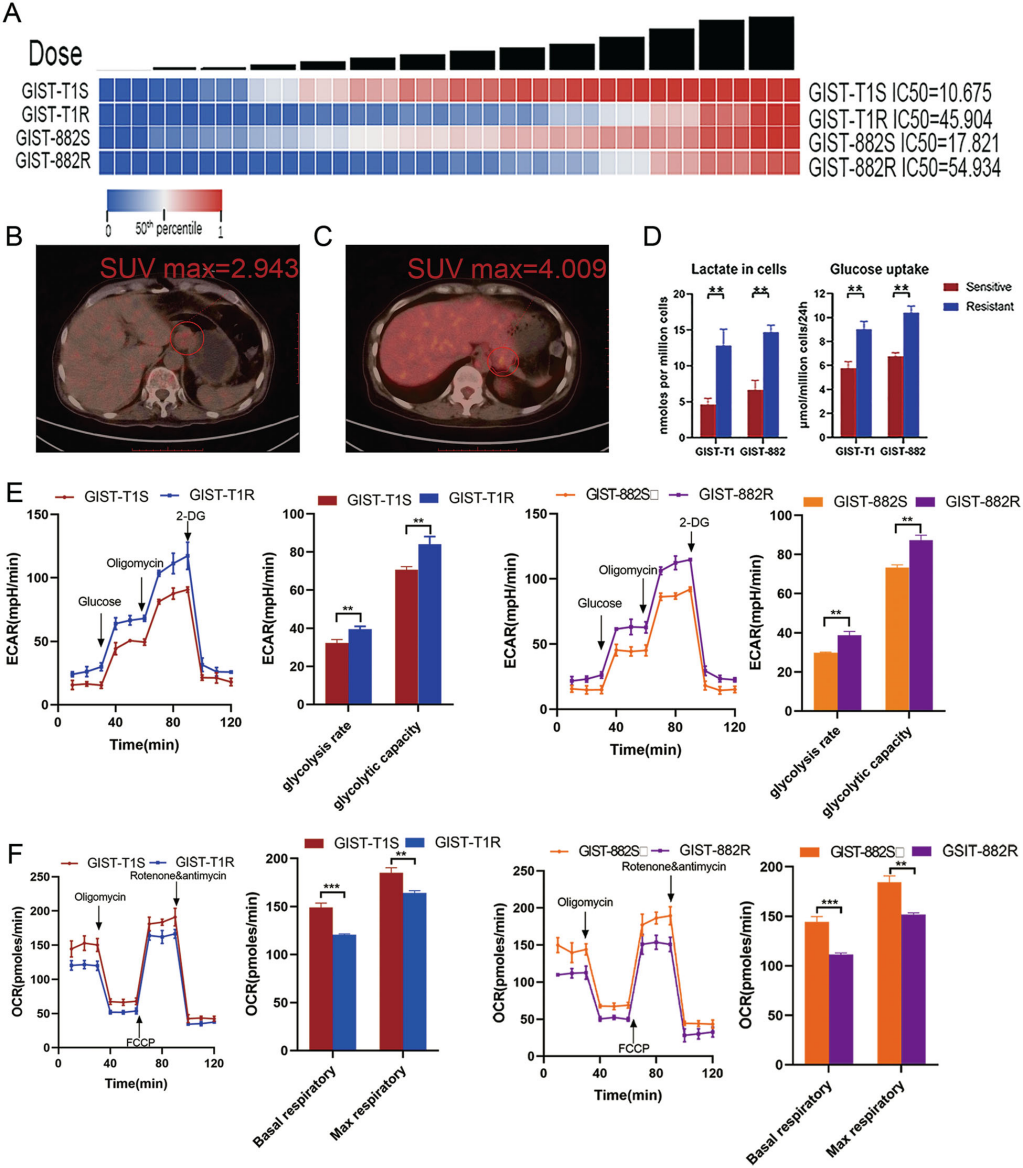
IM resistant cells have increased glycometabolism activity GIST. **a** Heatmaps representing cell viability after treatment of IM. **b** 18FDG-PET-CT performed in a 52-year-old man. Transaxial fused image showing mild tracer uptake in the tumor (abnormal finding, circled). **c** 18FDG-PET-CT performed in a 58-year-old man who relapsed after complete resection of tumor 10 months ago and treatment of IM 400 mg/day for 9 months. Transaxial fused image showing focal tracer uptake in the tumor (abnormal finding, circled). **d** Lactate, and glucose uptake analysis of GIST cell lines were determined via flow cytometry and the colorimetric method, respectively. **e** Analysis of extracellular acidification rate of GIST-T1 cells (left) and GIST-882 cells (right). The extracellular acidification rate after glucose treatment indicates the rate of glycolysis. The extracellular acidification rate after oligomycin treatment indicates the glycolytic capacity. **f** Analysis of oxygen consumption rate of GIST-T1 cells (left) and GIST-882 cells (right). The oxygen consumption rate before oligomycin treatment indicates the basal respiratory rate. The oxygen consumption rate after FCCP treatment indicates the maximum respiratory rate. The error bars represent the mean (*n* = 3) ± S.D. *P < 0.05, **P < 0.01, ***P < 0.001.

To obtain cells that can survive persistently exposure to imatinib, we continuously cultured GIST cells (T1 and 882) in medium containing IM until the outgrowth of a resistant population was obtained. After 2 years of continuous screening, we observed that GIST-T1 and GIST-882 cell lines both had statistically significant changes in IC50, indicating that we established stable resistant GIST cell lines,GIST-T1R and GIST-882R (Fig. 1a).

In the course of culturing the resistant cell lines, we found that in GIST-T1R and GIST-882R cells, the color of the culture medium changed from pink to orange more rapidly and the pH of the medium decreased from 7.7 to 6.4 at day 3 (Supplementary Fig. 1A). In addition, CCK-8 assays were performed to found that optical density (OD) values displayed no changes at day 3, excluding the possibility that the changes of pH caused by the difference of cell numbers (Supplementary Fig. 1B, C). Furthermore, we were surprised that in 11 patients who accepted 18F-fluorodeoxyglucose-positron emission tomography/computed tomography (18FDG-PET/CT) examination, eight were positive and three negative (Table 1). Three of five sensitive lesions were positive(Max SUV, 2.943–3.102, Fig. 1b) while five of six resistant were positive (Max SUV, 3.812–4.009, Fig. 1c). Based on this observation, we hypothesized that resistant cell lines might have more activated glycolysis and subsequently lactate production than sensitive cells. As expected, resistant cells utilized more glucose and produced more lactate (Fig. 1d). ATP content of resistant cells was also higher than sensitive cells (Supplementary Fig. 1D). To verify this change of resistant cells on glycolysis and oxidative phosphorylation, extracellular acidification rates (ECARs) and oxygen consumption rates (OCR) of GIST cell lines were measured. Resistant cells have increased glycolytic capacity and glycolysis rate (Fig. 1e). Moreover, the mitochondrial function of oxidative phosphorylation indicated by the alternation in oxygen consumption and respiratory capacity was also changed. Basal and maximum OCR were both higher in sensitive cells compared with resistant cells (Fig. 1f). These data collectively suggest that resistant cells occurred a greatly cellular metabolic shift in glycometabolism.

**Table 1:** Clinicopathological characteristics of 48 patients with GIST.

Characteristics		Number	No. of patients		*P*-value
			Sensitive patients	Resistant patients	
Age (years)	≥60	30	17	13	0.1387
	<60	18	14	4	
Gender	Male	20	11	9	0.2407
	Female	28	20	8	
Tumor size	>=5 cm	16	9	7	0.3933
	<5 cm	32	22	10	
Risk stratification	High risk	48	31	17	—
Primary mutation	KIT Exon 11	48	31	17	—
Secondary mutation	Positive		0	0	—
	Negative		0	11	
	NA		31	6	
Imatinib dose (mg/day)	400	48	31	17	—
Primary localization	Stomach	33	21	12	0.8388
	Small intestine	15	10	5	
Recurrence localization	Liver	10	0	10	—
	Stomach	5	0	5	
	Small intestine	2	0	2	
18FDG-PET/CT	Positive	8	3	5	—
	Negative	3	2	1	
	NA	37	26	11	

*NA* not available.

### IM-resistant cells display activation of PGD in PPP

**Fig. 2:**
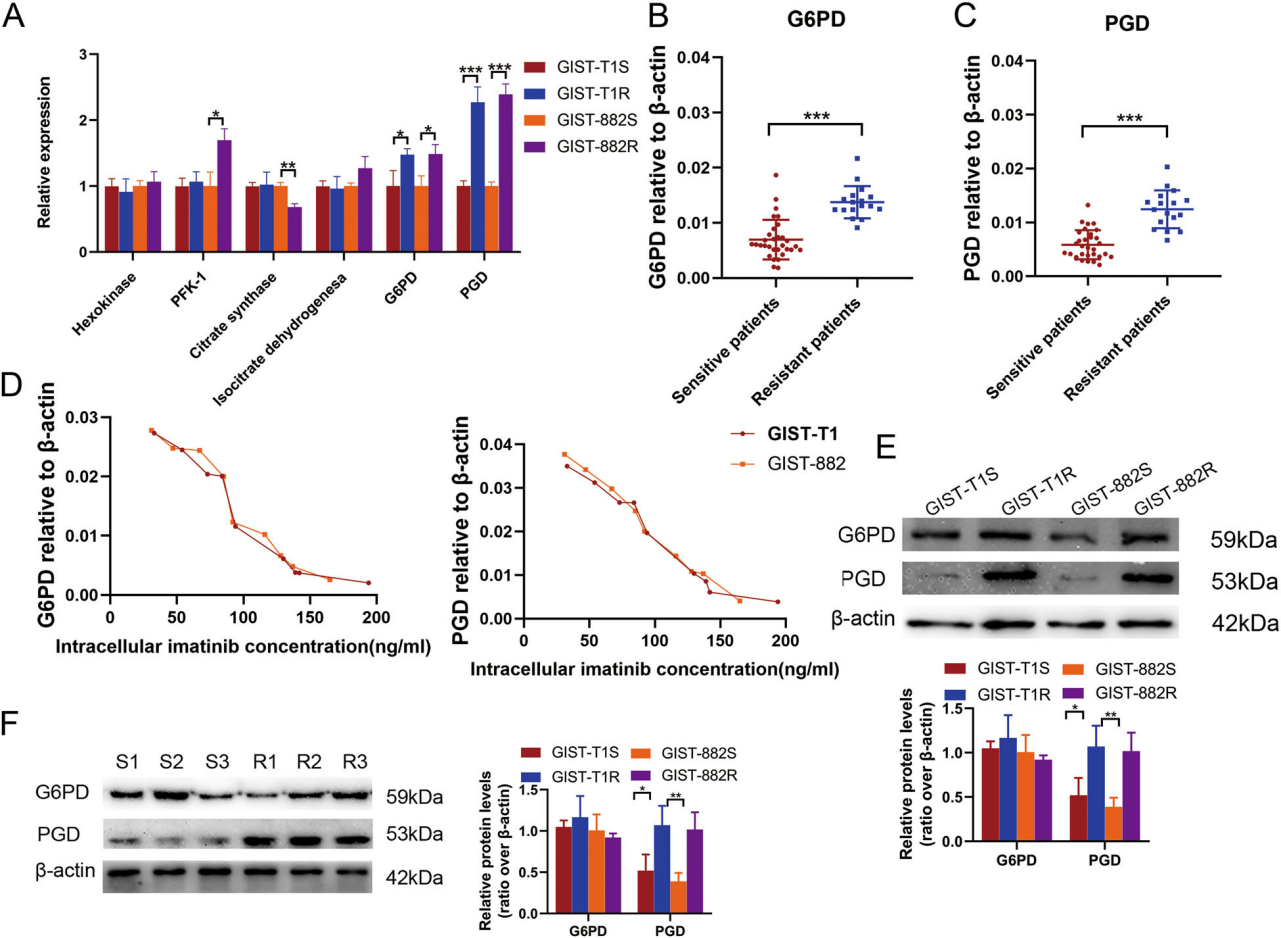
PGD expression is frequently upregulated in resistant cells and tissues in GIST. **a** qRT-PCR analysis of the expression of key enzymes in glycometabolism pathway in sensitive and resistant cell lines. **b** PGD expression level in sensitive and resistant tissues of GIST analyzed by qRT-PCR. **c** G6PD expression level in sensitive and resistant tissues of GIST analyzed by qRT-PCR. **d** Dot plot demonstrating the correlation between the IM intracellular concentration and G6PD and PGD expression level in GIST cells with various resistant capacity analyzed by qRT-PCR. **e** Levels of PGD and G6PD protein in GIST cells. **f** Levels of PGD and G6PD protein in GIST tissues. The error bars represent the mean (*n* = 3) ± S.D. *P < 0.05, **P < 0.01, ***P < 0.001.

We first measured key regulatory enzymes expression in glucose metabolism, including hexokinase (HK), 6-phosphofructokinase-1 (PFK-1), citrate synthase(CS), isocitrate dehydrogenesa (IDH), glucose-6-phosphate dehydrogenase (G6PD) and phosphogluconate dehydrogenase(PGD), using quantitative reverse transcriptase-PCR (qRT-PCR) in GIST-T1 and GIST-882 cell lines and 31 sensitive and 17 resistant tumor tissues from GIST patients. The results revealed that the expression of PGD and G6PD in resistant cells and tissues were both significantly higher than sensitives while the other enzymes did not present consistent trend (Fig. 2a and Supplementary Fig. 2). The PGD and G6PD expression in resistant tissues were 2.13-fold and 1.98-fold higher than sensitive GIST tissues, respectively (*P* < 0.001) (Fig. 2b, c). The clinicopathologic features of the GIST patients are summarized in Table 1.

To better explore the relationship between resistant capacity of cell lines and the expression of PGD and G6PD, we detected the intracellular IM concentration in GIST cell lines with different response to IM. As shown in Fig. 2d, the expression of PGD and G6PD had negative correlation with intracellular IM concentration in both T1 and 882 cells. However, only the expression of PGD showed consistent upregulation at protein level with transcriptional level both in cells and tissues while G6PD presenting no significant difference, indicating that the abnormal upregulation of the key regulatory enzyme, PGD, in PPP is possible to be one of causes in drug resistance (Fig. 2e, f).

### Activation of PPP reverse cell cycle arrest and apoptosis via reducing IM-inducing ROS

**Fig. 3:**
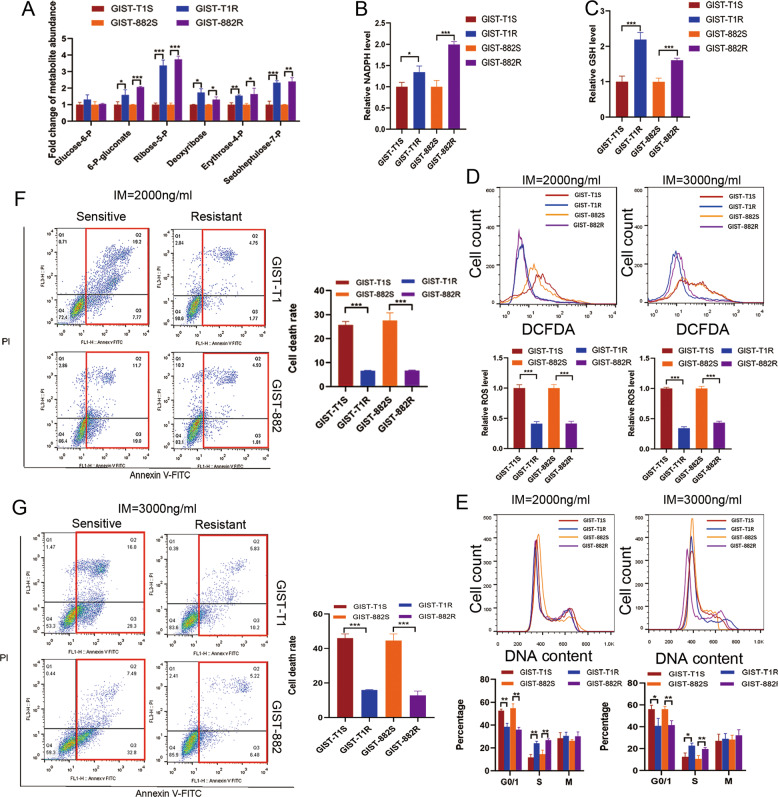
Analysis of the effect of PGD in PPP on ROS production and cell cycle arrest induce by IM in GIST. **a** Targeted analysis of abundance of different metabolites in PPP. **b** NADPH levels in GIST cells. **c** GSH levels in GIST cells. **d** FACS analysis (above) and statistical results (below) of ROS levels after treatment with 2000 ng/ml or 3000 ng/ml for 24 h. **e** DNA content analysis of GIST cells after exposed to IM with 2000 ng/ml or 3000 ng/ml for 24 h. **f** FACS analysis of cell apoptosis after exposed to IM with 2000 ng/ml for 24 h. **g** FACS analysis of cell apoptosis after exposed to IM with 3000 ng/ml for 24 h. The error bars represent the mean (*n* = 3) ± S.D. *P < 0.05, **P < 0.01, ***P < 0.001.

To further examine the impact of PGD on PPP, we subjected cell extracts (GIST-T1S, GIST-T1R, GIST-882S, GIST-882R) to LC–MS analysis to determine the relative enrichment of specific metabolites. Figure 3a demonstrated a general increase in PPP metabolites in resistant cell lines as compared with sensitive cells. Consistent with increased glucose flux through the PPP, resistant cells increased NADPH levels and glutathione (GSH) levels (Fig. 3b, c).

To further confirm that PGD plays an important role in antioxidant defense, we measured ROS production, cell cycle and apoptosis rate of cells in 2000 ng/ml and 3000 ng/ml concentration of IM. The level of ROS induced by IM was significantly suppressed in resistant cells compared with sensitive cells in both two concentration (Fig. 3d). Because ROS is also a potent inducer of the antioxidant defense machinery, we examined the expression of NRF2, GPX3, and TRX1 of GIST cell lines added with IM. Supplementary Fig. 2E showed that there was no significant difference in their expression. The induction of ROS has been reported to be related to tumor progression, and survival^21^. As shown in Fig. 3e, sensitive cells had larger percentage of cells in G1 phase and smaller percentage in S phase. Moreover, sensitive cells exhibited a higher rate of apoptosis compared with resistant cells, while the same trend was observed when IM concentration increased from 2000 ng/ml to 3000 ng/ml (Fig. 3f, g). Based on these findings, we hypothesized that through accelerating PPP, PGD rescued cell apoptosis in GIST cells caused by ROS induced by IM.

### ROS reverse the cell resistance with high expression of PGD in vitro

**Fig. 4:**
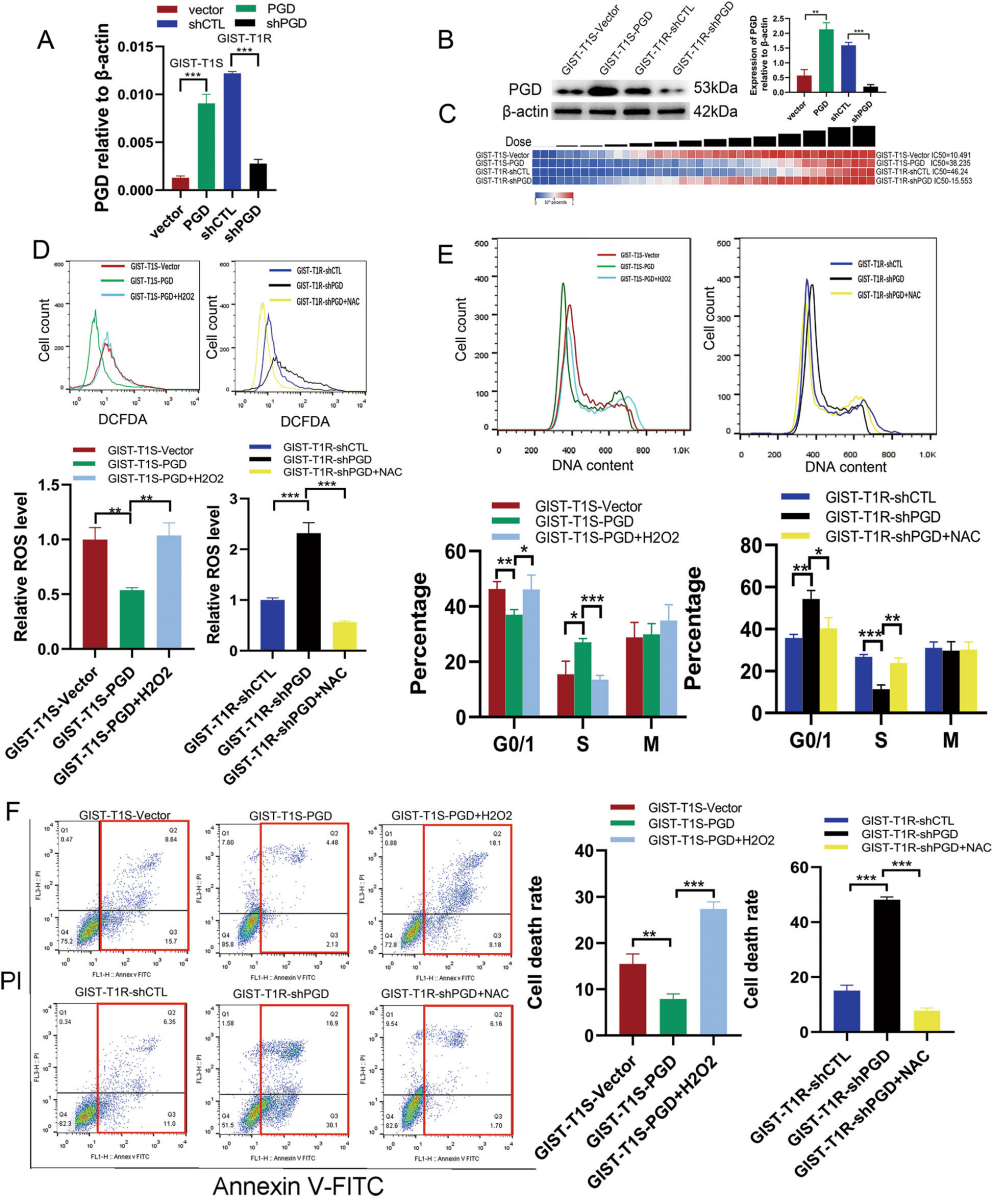
ROS reverse the resistance of GIST cells with high expression of PGD in vitro. **a** PGD expression levels were evaluated by qRT-PCR after PGD overexpression and knockdown in GIST-T1S and GIST-T1R cells, respectively. **b** PGD expression levels were evaluated by WB after PGD overexpression and knockdown in GIST-T1S and GIST-T1R cells, respectively. **c** Resistant capacity were evaluated by IC50 after PGD overexpression and knockdown in GIST-T1S and GIST-T1R cells, respectively. **d** FACS analysis (above) and statistical results (below) of ROS levels of GIST-T1S-vector, T1S-PGD, T1R-shCTL, T1R-shPGD, 10 mM NAC or 20 μM H2O2 for 24 h. **e** DNA content analysis (above) and statistical results (below) of GIST-T1S-vector, T1S-PGD, T1R-shCTL, T1R-shPGD, 10 mM NAC or 20 μM H2O2 for 24 h. **f** FACS analysis of cell apoptosis (above) and statistical results (below) of GIST-T1S-vector, T1S-PGD, T1R-shCTL, T1R-shPGD, 10 mM NAC or 20 μM H2O2 for 24 h. The error bars represent the mean (*n* = 3) ± S.D. *P < 0.05, **P < 0.01, ***P < 0.001.

To determine the impact of PGD on GIST cells, we elevated PGD expression in GIST-T1S and GIST-882S cells, and repressed its expression in GIST-T1R and GIST-882R cells by lentivirus infection and verified the altered expression of PGD in these GIST cell lines by qRT-PCR and western blotting (Fig. 4a, b and Supplementary Fig. 3A, B). IC50 changes shown in Fig. 4c and Supplementary Fig. 3C mean that PGD played a crucial role in the development of drug resistance.

As expected, PGD knockdown in resistant cells obvious elevated ROS level while PGD overexpression in sensitive cells resulting in decreased ROS level (Fig. 4d and Supplementary 3D). However, these changes could be rescued by treatment with the antioxidant N-acetylcysteine (NAC) and H2O2 (20 μM), respectively (Fig. 4d and Supplementary 3D). We further analyzed whether the overexpression of PGD suppressed cell cycle arrest and apoptosis in resistant cells through decreasing ROS. As shown in Fig. 4e, f and Supplementary 3E, F, PGD overexpression in GIST cells significantly increased the percentage of cells in S phase and decreased the cell apoptosis. To confirm that, we also performed rescue experiment. The attenuated cell cycle arrest and cell apoptosis induced by PGD overexpression could be corrected by H2O2; also, abnormal large amount of cells in G1 phase and cell death caused by PGD knockdown could be weakened by NAC (Fig. 4e, f and Supplementary 3E, F). Taken together, these results show that upregulation of PGD could reverse cell cycle arrest and apoptosis of GIST cells resulted by IM-inducing ROS.

### PGD is a direct functional target of HIF-1α in GIST cells

**Fig. 5:**
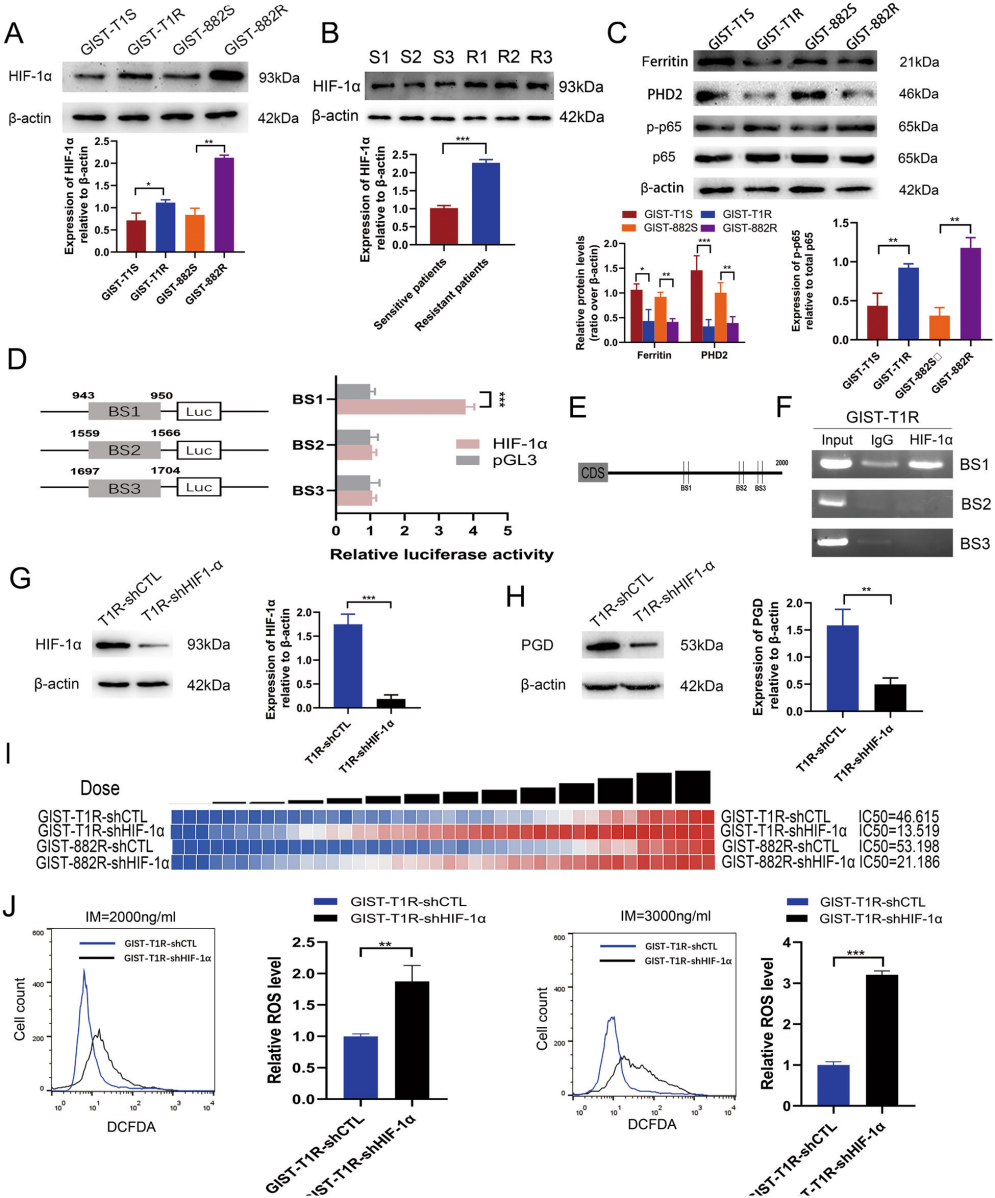
Upregulation of HIF-1α promotes IM resistance of GIST cells via binding to the promoter region of PGD gene. **a** Levels of HIF-1α protein in GIST cells. **b** Levels of HIF-1α protein in GIST tissues. **c** Levels of ferritin, PHD2, p-p65 and p65 protein in GIST cells. **d** Schematic representation of the luciferase plasmid with BS1, BS2, and BS3 in promoter region of PGD gene (left) and a luciferase reporter assay was used to evaluate interactions between HIF-1α and the targeting sites of PGD gene (right). **e** Schematic diagram exhibiting three potential HIF-1α binding sites in promoter region of PGD gene. **f** ChIP analysis was performed in GIST-T1R cells. A relatively brighter band was detected in HIF-1α compared with the control IgG with BS1. No band was observed with BS2 and BS3. **g** HIF-1α expression levels were evaluated by WB after HIF-1α knockdown in GIST-T1R cells. **h** Levels of PGD protein in GIST-T1 cells with HIF-1α knockdown. **i** Resistant capacity was evaluated by IC50 after HIF-1α knockdown in GIST cells. **j** FACS analysis (above) and statistical results (below) of ROS levels of GIST-T1 cells with HIF-1α knockdown. The error bars represent the mean (*n* = 3) ± S.D. *P < 0.05, **P < 0.01, ***P < 0.001.

HIF-1α is known to be required to allow cells to secrete excess glycolytic pyruvate as lactate^22–24^. Based on our experiment results that the ECAR of resistant cells was higher than sensitive cells in GIST, we supposed HIF-1α may be upregulated and a key contributor in drug resistance in GIST cells. Thus, we examined the expression of HIF-1α in cells and tissues. Increased expression of HIF-1α at protein level was observed in both resistant cell lines and tissues (Fig. 5a, b). We also examined some factors closely related to the expression of HIF-1α-PHD2, ferritin and NF-kB^25–27^. Results showed that the expression of PHD2 and ferritin were lower in resistant cell lines while NF-kB was higher in resistant cell lines, contributing to the high level of HIF-1α in resistant cells (Fig. 5c).

Several studies showed that HIF-1α is one of most common transcription factors. Therefore, to investigate whether PGD is a transcription target of HIF-1α in GIST cells, we carried on luciferase reporter assay containing predicted HIF-1α binding sites on PGD promoter. We analyzed 0–2000 bp upstream regions of PGD transcription start site. Jaspar (http://jaspar.genereg.net) was used to locate the putative HIF-1α binding sites (BS) within the 2000 bp PGD promoter region. We found 9 predicted binding sites for HIF-1α on PGD promoter. According to the score of each binding site, we chose the top three binding sites in the sequence. We designed three pGL3-based luciferase reporter constructs (BS1–BS3) containing potential binding areas (Fig. 5d). The luciferase reporter assay data revealed that HIF-1α overexpression significantly increased BS1 luciferase reporter activity, compared with empty vector control (Fig. 5d, *P* < 0.001). Of note, we did not detect increased luciferase reporter activity of BS2 or BS3, following similar conditions (Fig. 5d). To further probe the direct HIF-1α binding site to the PGD promoter region within the natural chromatin context of GIST cells, a ChIP assay was performed by using three sets of primers each containing a binding site (Fig. 5e). HIF-1α showed a stronger ability to associate with BS1 in the promoter region of PGD gene in GIST-T1R and GIST-882R cells (Fig. 5f and Supplementary Fig. 4A). These results strongly suggest the presence of functional HIF-1α binding site on PGD promoter, localized in BS1 of PGD, playing an active role in GIST cells.

To better understand the influence of HIF-1α on PGD expression in GIST cells, we downregulated HIF-1α expression in GIST-T1R and GIST-882R cells by lentivirus infection and verified the altered expression of HIF-1α in these GIST cell lines by western blotting (Fig. 5g and Supplementary Fig. 4B). Firstly, we found that loss of HIF-1α expression significantly suppressed the expression of PGD using Western Blot (Fig. 5h and Supplementary Fig. 4C). Furthermore, HIF-1α’s function in drug resistance was analyzed and found to be consistent with PGD. GIST-T1R and GIST-882R cells with HIF-1αknockdown markedly decreased the capacity of IM tolerance (Fig. 5i). Finally, loss of HIF-1α expression in GIST resistant cells markedly promoted the ROS level (Fig. 5j and Supplementary Fig. 4D). Taken together, these results demonstrated that HIF-1α upregulated PGD causing the activation of PPP and resistance of GIST cells.

### HIF-1α and PGD result in IM-resistance in vivo

**Fig. 6:**
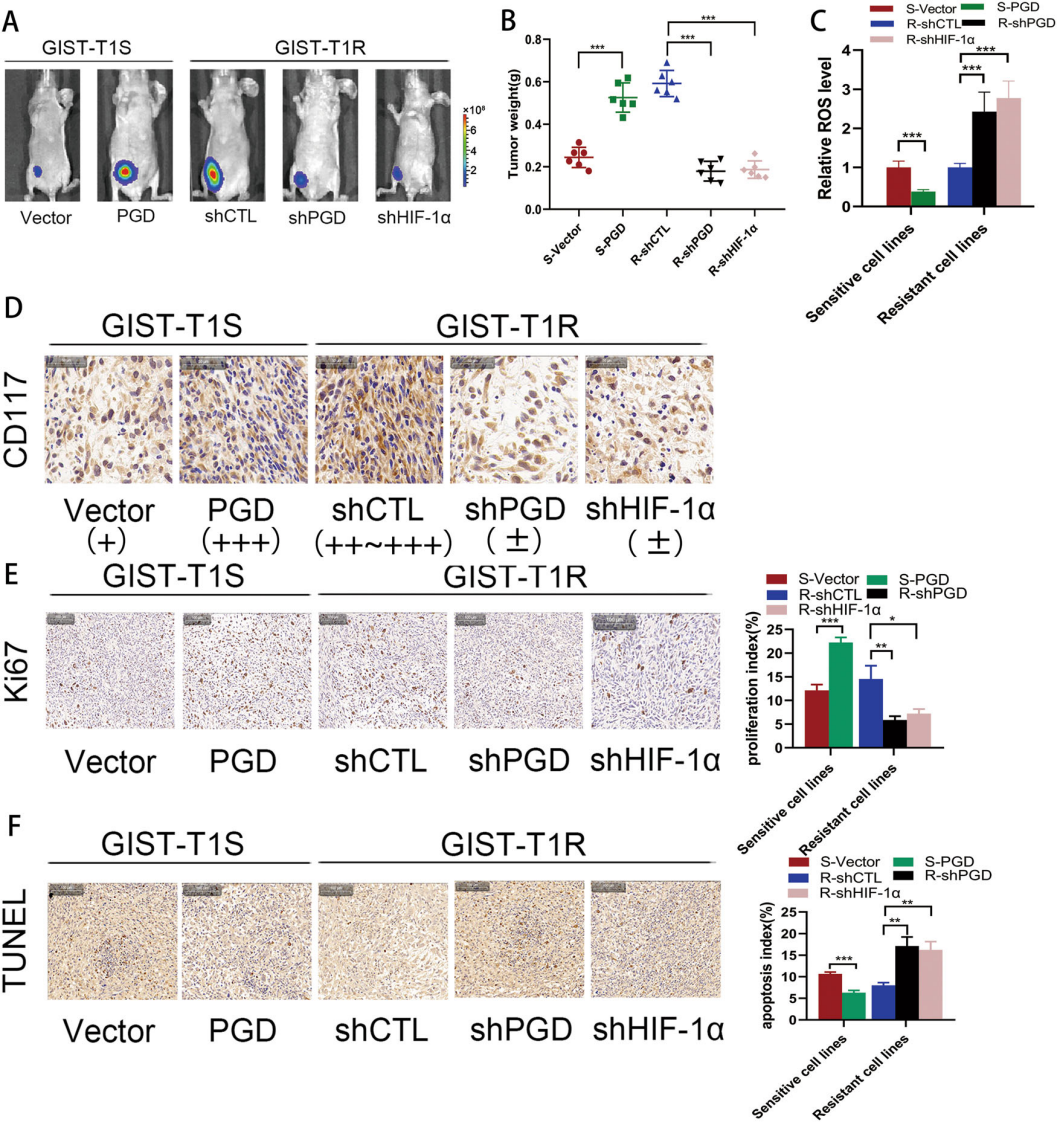
HIF-1α promotes drug resistance in vivo. **a** Bioluminescent images of mice administered with GIST cells. Images were taken after 8 weeks. *n* = 3 per group. **b** The in vivo effect of HIF-1α and PGD was evaluated in xenograft mouse models bearing tumors originating from GIST-T1 and GIST-882 cells; *n* = 3 per group. **c** ROS levels were measured in xenograft mouse models bearing tumors originating from GIST-T1 and GIST-882 cells; *n* = 3 per group. **d** Representative CD117 staining of primary tumor tissues. **e** Representative Ki67 staining of primary tumor tissues. **f** TUNEL assay were used to determine the effects of HIF-1α and PGD expression alteration on cell apoptosis in the samples collected from nude mice. The error bars represent the mean (*n* = 3) ± S.D. *P < 0.05, **P < 0.01, ***P < 0.001.

To better investigate the effects of HIF-1α and PGD on drug resistance in vivo, cells transfected with the vectors mentioned above were injected into the flanks of nude mice drinking water containing IM for 2 weeks at the same time to generate tumors ectopically. Eight weeks later, as shown in Fig. 6a, the tumor progression was faster in resistant group (T1R-shCTL, 882R-shCTL, T1S-PGD, 882S-PGD) compared with sensitive group (T1S-vector, 882S-vector, T1R-shPGD, 882R-shPGD, T1R-shHIF-1α, 882R-shHIF-1α). It was accordant in Fig. 6b that the tumor weight in resistant group were heavier. Furthermore, we measured ROS in tumor tissues, and the results had the same trend as in cell lines (Fig. 6c). Immunohistochemical analyses of the implanted tumors in mice revealed significant downregulation of CD117 expression in sensitive group compared with that in resistant group (Fig. 6d and Supplementary Fig. 5A). Subsequently, Ki-67 staining and TUNEL assays were used to further verify that PGD and HIF-1α suppressed apoptosis and increased resistance to IM. The distribution of Ki-67 revealed a reduced proliferation index in those sensitive group compared with that of the resistant group (Fig. 6e and Supplementary Fig. 5B). TUNEL assays revealed a dramatically higher apoptosis index in the tumors of sensitive group compared with that in resistant group (Fig. 6f and Supplementary Fig. 5C). These in vivo data strongly indicate the role of PGD and HIF-1α as causes of IM resistance in GIST.

## Discussion

Recently, dramatic changes of metabolic phenotypes in many tumors have been noticed including lung cancer, breast cancer, ovarian cancer, melanoma and osteosarcoma^28–32^. Especially, the activation of PPP is implicated in the development of various tumors^33,34^. However, in terms of GIST, how sustained tyrosine kinase inhibition has association with those metabolic changes and then cause drug resistance is still vague. Further exploration of the clinical significance, biological effects and potential mechanisms by which specific metabolic phenotype affect GIST and its resistance is needed. Surprisingly, in this study, we show that IM resistant cells displayed an increase in glycolysis. Moreover, we find that PPP activity was strongly higher in resistant cells and tumor tissues and that forced PPP activity was resulted by PGD overexpression. These observations indicate that PGD probably acts as a contributor of IM resistance in GIST.

Previous studies have demonstrated that IM may induce ROS generation in tumor cells to cause cell dormancy, apoptosis and death^35–38^. Interestingly, one of the most important production of PPP is NADPH, a crucial antioxidant that provides reducing power to the glutathione and thioredoxin systems^39^, the dysregulation of which may influence the cellular ROS level. In this study, we demonstrated that the PGD regulates PPP and ROS levels, resulting in deceased G1 phase arrest and apoptosis of GIST cells exposed to IM.

HIF-1α, an important transcription factor, has been largely studied for its involvement in crucial aspects of hypoxia and ischemia, inflammation, and cell survival^40,41^. The regulation of HIF-1α is a complex process and involves a number of molecules and pathways, among which the most direct regulatory way is the impact of ROS on HIF-1α^21,42,43^. Further research and discovery regarding HIF-1α regulation by oxidative stress is warranted for better understanding of disease development and potential therapeutics for pathologies in tumor. It has been reported that in CML, HIF-1α is required for the survival and proliferation of IM-resistant cells and its activation can promote viability in cells^44,45^. To further explore the exact mechanism of HIF-1α and PGD involved in this resistant effect, we postulated that the transcription factor HIF-1α might have interaction with PGD. Here, we showed that when HIF-1α binds with PGD promoter sequence, the PGD expression and PPP activity are increased, stimulating the progression of GIST cells from the G1 to the S phase of the cell cycle. When PGD overexpression GIST cells exposed to H202, they become sensitive to IM again and produce more ROS, leading to more cells staying in G0/G1 phase and experiencing apoptosis. Thus, our study demonstrates an essential role of HIF-1α in the metabolic regulation and thus highlights a key role of HIF-1α in the integration of IM resistance in GIST cells.

**Fig. 7:**
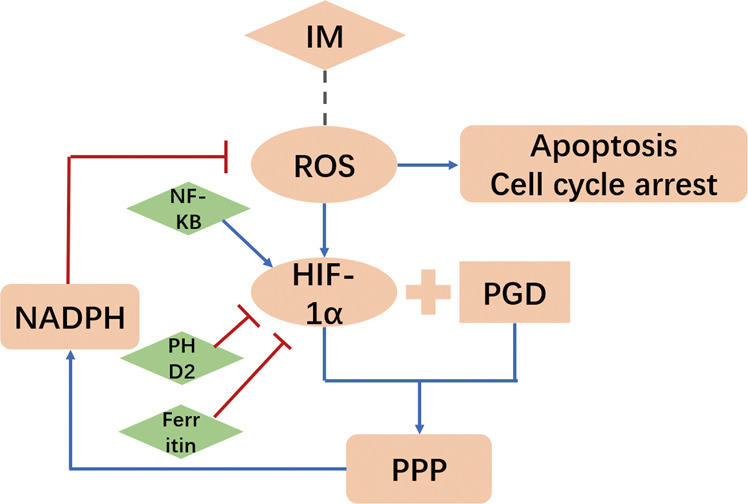
Schematic for regulation of cellular metabolism by the HIF-1α–PGD axis in GIST. Blue arrows means acceleration. Red lines means inhibition.

In conclusion, we demonstrate that PGD plays critical roles in GIST cell resistance. The HIF-1α-PGD-PPP axis inhibits tumor apoptosis mainly through metabolic reprogramming (Fig. 7). In this process, the ROS level is also significantly affected. These observations provide new evidence for an interplay between drug resistance and cell metabolism. The impact of IM on ROS levels could be partially abrogated via modulation of PPP, emphasizing the crucial effects of reprogramming metabolism in tumor progression and drug tolerance. Inhibiting PPP not only compromises the metabolism of remaining tumor cells, but also drives tumor cells toward a sensitive phenotype which ultimately increases the effects of IM.

## Material and methods

### Cell culture

The human GIST-T1 cell line, harboring deletion mutation in KIT exon 11, and GIST-882, carrying missense mutation in KIT exon 13, whose mutations were validated via next generation sequencing technology, were purchased from Biowit Technologies (Shenzhen, China). Cells were cultured in complete DMEM medium with 15% fetal bovine serum (FBS) and 1% Penicillin-Streptomycin Solution in a humidified chamber with 5% CO_2_ at 37 °C.

### Resistant cell lines establishment

IM-resistant cell lines, GIST-T1R and GIST-882R, were obtained from sensitive parental GIST-T1 and GIST-882. To establish IM resistant cell lines, drug-containing medium with intermittently increasing IM (Glivec, Novartis AG, Switzerland) concentrations (from 5 μM and gradually increasing to 50 μM) was added to culture medium when GIST cells in the exponential phase^46^. After culturing for 48 h, the IM-containing medium was replaced. The cells were then passaged when cell fusion. After two years of screening, we established stable resistant GIST cell lines (GIST-T1R and GIST-882R) successfully. Using next generation sequencing technology,we detected no secondary mutations in IM resistant cell lines.

### IC 50 and resistance index (RI) calculation

The CCK-8 assay (CK04, Dojindo, Japan) was performed to determine the IC50 and RI of GIST cell lines. Briefly, 5000 GIST cells were seeded in 96-well plate and cultured in 100 μl complete medium. After washed with phosphate-buffered saline (PBS) carefully, GIST-T1 and GIST-882 cells were exposed to fresh DMEM medium with 2.5, 5, 10, 15, 20, 25, 30, 35, 40, 50, 62.5, 75, and 80 μM IM for 48 h in humidified chamber. For each concentration, a blank well for each cell line was set up, as well as three duplicate wells. Next, each well was added 10 μl CCK-8 reagent and incubated for 2 h. Then automated micro plate reader was used to measure the OD value of each well at 450 nm.

### OCR/ECAR measurements

Metabolic analysis of GIST cells were determined using the analyzer Seahorse XF24 extracellular flux analyzer (Seahorse Biosciences) according to the manufacturer’s instruction. Briefly, 2 × 10^4^ cells were seeded in Seahorse plates, incubated at 37 °C in 5% CO_2_ overnight and washed in Seahorse buffer. Then, 175 μl of Seahorse buffer and 25 μl each of glucose (10 mmol/l), oligomycin (1 mmol/l), and 2-deoxyglucose (100 mmol/l) were added to determine the ECAR. To measure the OCR, 175 μl of Seahorse buffer containing 25 μl each of oligomycin (1 mmol/l), carbonyl cyanide-4-(trifluoromethoxy) phenylhydrazone (FCCP) (2 mmol/l), and antimycin/rotenone (0.5 mmol/l) was automatically injected into the analyzer.

### Glucose uptake and lactate assay

Glucose uptake assays were performed using 2-NBDG (11046, Cayman). Cells were incubated in 100 μM 2-NBDG for 30 min. And then, cells were washed and resuspended in ice-cold PBS. We collected cells and analyzed their glucose uptake cytofluorometrically by recording FL-1 fluorescence. For analysis of lactate concentration, a lactate assay kit (K627, BioVision) was used according to the manufacturer’s instructions.

### Patients and tissue

Sensitive GIST samples were obtained from 31 patients who underwent radical resection for GIST at the First Affiliated Hospital of Nanjing Medical University China, from February 2015 to February 2018. All patients accepted IM treatment after surgery and none of them suffered from recurrent tumors after surgery for at least 1 year. As for resistant samples, tumor tissues were obtained through CT-guide biopsy or surgery from 17 patients having disease progression even though they taking IM regularly. All tissues were put into liquid nitrogen immediately after isolation. Prior consent from the patients and approval from the Ethics Committee of the First Affiliated Hospital of Nanjing Medical University have been acquired. In all cases, two experienced pathologists had conformed the diagnosis and grading.

### RNA extraction and qRT-PCR

Total RNA was extracted from cell lines and frozen tissues using TRIzol reagent (15596018, Invitrogen) and was reverse transcribed into complementary DNA (cDNA) using PrimeScript RT Master Mix Kit (RR036A, TaKaRa), which was then amplified using Universal SYBR Green Master Mix (4913914001, Roche). Gene expression was normalized to β-actin gene. The sequences of primers used are listed in Supplementary Table 1.

### IM incubation and IM concentration detection

We constantly added medium with IM in 2.5, 5, 10, 15, 20, 25, 30, 40, and 50 μM concentration to GIST-T1 and GIST-882 cell lines for at least 2 months to establish cell lines with various tolerance to IM (2.5T1R and 882R, 5T1R and 882R, 10T1R and 882R, 15T1R and 882R, 20T1R and 882R, 25T1R and882R, 30T1R and 882R, 40T1R and 882R, 50T1R and 882R).

To detect the intracellular concentration of IM, culture medium of GIST-T1, GIST-882 cells with various resistance was replaced by medium containing 3000 ng/ml IM after washed carefully with (PBS). After incubation for 24 h at 5% CO_2_ in 37 °C, cell lines were washed softly with ice-cold PBS for 3 times. After detached from each well by 1 ml trypsin/EDTA, cells from each well were collected, counted, and centrifuged for 5 min at 1000 rpm. Those deposits were resuspended by PBS to make the final concentration was 1 × 10^6^ cell/100 μl. After resuspension, the cells were lysed by ultrasound tested as previously described^47^.

### Antibodies and reagents

The following antibodies were used: PGD (ab129199), G6PD (ab210702), HIF-1α (ab243860), β-actin (ab8226), Ki-67 (ab156956), CD117(ab32363) from Abcam. The following reagents were used: NAC (A9165), H2O2 (88597) from Sigma-Aldrich.

### Western blotting

Total protein was extracted using a protein extraction kit (KGP9100, Key Gene). Equal amounts of protein were added into 10% gels by sodium dodecyl sulfate–polyacrylamide gel electrophoresis and transferred onto the polyvinylidene fluoride (PVDF) membrane. After blocking in a mixture of 5% bovine serum albumin (BSA) in Tris-buffered saline and Tween-20 (TBST) buffer, the membranes were incubated with specific primary antibodies at 4 °C overnight followed by secondary antibodies at room temperature for 2 h. Protein expression levels were visualized by the HRP Substrate (WBKL0100, Millipore) and an enhanced chemiluminescence (ECL) detection system.

### Determination of NADPH and GSH levels

The colorimetric NADP^+^/NADPH Quantitation Kit (BioVision) was applied to determine NADPH levels according to the manufacturer’s protocol. The 450 nm signal was recorded using the Victor 3 microplate reader and then normalized to protein concentration. The Glutathione Assay Fluorimetric Kit (Sigma-Aldrich) was used to measure GSH levels according to the manufacturer’s procedure. The signal was recorded on the Victor 3 microplate reader and then normalized to protein concentration.

### LC–MS for metabolite analysis

The profiling of representative metabolites in PPP were carried out on the Xevo TQ-S tandem mass spectrometer, equipped with an electrospray source which operates in the negative-ion multiple-reaction monitoring mode. The ion source settings were as below: source temperature, 120 °C; ion spray voltage, −3500 V; cone gas, desolvation gas and nebulizer gas at settings 150 lh^−1^, 600 lh^−1^, and 7 Bar, respectively. Chromatographic separation was performed on a 5 μm, 100 × 2.0 mm Phenomenex Luna Amide column. The quantitative multiple-reaction monitoring transition of particular metabolite was described as follows, G6P: *m*/*z* 259497; 6-phosphogluconate: *m*/*z* 275479; ribose-5-phosphate: *m*/*z* 229497; erythrose-4-phosphate: *m*/*z* 199497; sedoheptulose-7-phosphate *m*/*z* 289497. Data were processed using MassLynx software (Version V4.1). Peak areas of each metabolites were normalized to the total protein amount. The fold changes of the relative level of targeted metabolites are calculated.

### Cell cycle, apoptosis, and ROS level analyses

Cell cycle analysis was conducted with cells more than 10,000 stained with propidium iodide (PI) by fluorescence activated cell sorter (FACS). Cell apoptosis was detected by FACS with cells stained with PI and Annexin V-FITC (559763, BD Pharmingen) according to the manufacturer’s instructions and. Intracellular ROS levels were also examined by FACS of cells stained with DCFDA (S0033, Beyotime). For tissues, 5 μM DCFDA was applied to fresh tissues which were already washed by PBS for three times and incubated at 37 °C for 30 min. NIS-Elements was used to quantify the fluorescence intensity was quantified by the software.

### Lentivirus transfection

HIF-1α shRNA (Clone ID: NM_001530.x-3867s1c1), and PGD shRNA (NM_002631.2-941s21c1) in pLKO.1 vector (Genepharma, China) were packaged into lentivirus in HEK293T cells. Stable cell lines overexpressing PGD were established by lentiviral transduction (Genepharma, China) carrying the PGD DNA sequence. Stable cells were generated using puromycin.

### Chromatin immunoprecipitation assay (ChIP)

The ChIP assay was carried out by chromatin immunoprecipitation kit (17–371, EZ-ChIP, Millipore, Bedford, MA, USA) according to the manufacturer’s instructions. Briefly, cells were fixed with DNA by 37% formaldehyde, followed by adding 10× glycine solution. Chromatin fragments were sonicated into an average size of 500 bp using Bioruptor Pico (Diagenode, Denville, NJ) for 30 cycles (30 s On and 30 s Off at 40% amplitude). The immunoprecipitation antibody HIF-1αand control antibody normal mouse IgG, as well as protein A/G magnetic beads (CS204457, Millipore Sigma), were added into lysates and incubated at 4 °C overnight. Protein/DNA complexes were eluted, followed by DNA purification using wash buffers. Purified DNA was evaluated and analyzed by PCR. Specific primers were listed in the Supplementary Table 2.

### Luciferase reporter assay

Dual-Luciferase Reporter Assay System (E1910, Promega, Madison, WI, USA) was used to perform luciferase reporter assay. Briefly, an internal control, 5 ng of Renilla luciferase vector (pRL-TK; Promega), and 200 ng of a pGL3 reporter that contained various target regions were cotransfected into GIST cells. At 48 h after transfection, cells were harvested to measure the luciferase activity.

### Animal studies

For tumor growth assay, animals were divided randomly into ten groups which had six mice and a total of 4 × 10^6^ logarithmically growing GIST cells transfected with T1S-vector, T1S-PGD, T1R-shCTL, T1R-shPGD, T1R-shHIF-1α, 882S-vector, 882S-PGD, 882R-shCTL, 882R-shPGD, and 882R-shHIF-1α (*N* = 3 per group) in 100 μl PBS were injected into the flanks subcutaneously of female nude mice which were 4-week-old. Imatinib (600 mg/L in drinking water) was provided by Novartis lasting 2 weeks. After 8 weeks, the IVIS Imaging system (Caliper life Sciences, USA) was used to observe the tumor growth. Care of experimental animals was in accordance with Nanjing Medical University Institutional Animal Care and Use Committee.

### Immunochemical staining

All specimens used for immunochemical staining were fixed in 4% formalin and embedded in paraffin. The paraffin mass was cut into sections (thickness, 4 μm) that were mounted on slides and incubated with CD117 and ki-67 antibody at 4 °C overnight. Washed three times with PBS, the slides were incubated with HRP-polymer-conjugated secondary antibody at room temperature for 1 h. Finally, the slides were stained with a 3,3-diaminobenzidine solution for 3 min and counterstained with haematoxylin. The slides were examined in a blinded manner. Three fields on each slide were selected for examination, and the percentage of positive tumor cells and the cell-staining intensity in these fields were determined.

### TUNEL assay

Implanted tumors were fixed in 4% formalin, paraffinembedded and cut into 4-μm sections before HRP-conjugated dUTP staining. To detected apoptotic cells in the implanted tumors, a TUNEL apoptosis detection kit (Nanjing KeyGen Biotech, KGA7051, China) was used according to the manufacturer’s instructions. All sections were assessed under the microscope (Nikon, Japan). For individual group, the number of apoptotic cells and the total number of cells in five random fields were photographed and counted. The apoptotic index of the cancer cells was calculated using the following formula: Apoptotic index = apoptotic cells/total cells × 100%.

### Statistics analysis

All statistical analyses were performed using SPSS 20.0 software (SPSS Inc., Chicago, IL, USA); data are expressed as the mean ± S.D. *P* < 0.05 was considered statistically significant. Clinicopathological findings were compared using unpaired *t*-tests or Pearson *χ*2 Tests. The data obtained in cell line experiments and animal assays were subjected to Student’s *t*-test or one-way analysis of variance (ANOVA).

## Supplementary information

Supplementary information

Supplementary information 2

Supplementary information 3

Supplementary information 4

Supplementary information 5

Supplementary information 6
