# Supplementary material for: HIF-1α regulates cellular metabolism, and Imatinib resistance by targeting phosphogluconate dehydrogenase in gastrointestinal stromal tumors
Source: Cell Death Dis. 2020 Jul 27;11(7):586. doi: 10.1038/s41419-020-02768-4 (PMC7385157; doi:10.1038/s41419-020-02768-4)
Supplement: Supplementary file 1 — Supplementary information [file 41419_2020_2768_MOESM1_ESM.docx]

**Supplementary Figure legends**

**Figure S1**

**(A)** Color of the culture medium of GIST cells.

**(B)** Growth curves of GIST-T1 were recorded for 6 days, and the OD values were measured using a microplate reader at the recommended wavelength.

**(C)** Growth curves of GIST-882 were recorded for 6 days, and the OD values were measured using a microplate reader at the recommended wavelength.

**(D)** Relative ATP levels of GIST cell lines. The error bars represent the mean (n=3) ± S.D. * P < 0.05, ** P < 0.01, ***P < 0.001.

**Figure S2**

**(A)**HK expression level in sensitive and resistant tissues of GIST analyzed by qRT-PCR.

**(B)** PFK-1 expression level in sensitive and resistant tissues of GIST analyzed by qRT-PCR.

**(C)** CS expression level in sensitive and resistant tissues of GIST analyzed by qRT-PCR.

**(D)** IDH expression level in sensitive and resistant tissues of GIST analyzed by qRT-PCR.

**(E)** NRF2, GPX3 and TRX1 expression level evaluated by WB in sensitive and resistant GIST cell lines added with IM (3000ng/ml). The error bars represent the mean (n=3) ± S.D. * P < 0.05, ** P < 0.01, ***P < 0.001.

**Figure S3**

**(A)** PGD expression levels were evaluated by qRT-PCR after PGD overexpression and knockdown in GIST-882S and GIST-882R cells respectively.

**(B)** PGD expression levels were evaluated by WB after PGD overexpression and knockdown in GIST-882S and GIST-882R cells respectively.

**(C)** Resistant capacity were evaluated by IC50 after PGD overexpression and knockdown in GIST-882S and GIST-882R cells respectively.

**(D)** FACS analysis (left) and statistical results (right) of ROS levels of GIST-882S-vector,882S-PGD, 882R-shCTL,882R-shPGD, 10 mM NAC or 20 μM H2O2 for 24 h.

**(E)** DNA content analysis (above) and statistical results (below) of GIST-882S-vector,882S-PGD, 882R-shCTL,882R-shPGD, 10 mM NAC or 20 μM H2O2 for 24 h.

**(F)** FACS analysis of cell apoptosis (above) and statistical results (below) of GIST-882S-vector,882S-PGD, 882R-shCTL,882R-shPGD, 10 mM NAC or 20 μM H2O2 for 24 h. The error bars represent the mean (n = 3) ± S.D. *P < 0.05, **P < 0.01, ***P < 0.001.

**Figure S4**

**(A)** ChIP analysis was performed in GIST-882R cells. A relatively brighter band was detected in HIF-1α compared with the control IgG with BS1. No band was observed with BS2 and BS3.

**(B)** HIF-1α expression levels decreased by WB after HIF-1α knockdown in GIST-882R cells.

**(C)** Levels of PGD protein in GIST-882 cells with HIF-1α knockdown.

**(D)** FACS analysis (above) and statistical results (below) of ROS levels of GIST-T1 cells with HIF-1α knockdown. The error bars represent the mean (n = 3) ± S.D. *P < 0.05, **P < 0.01, ***P < 0.001.

**Figure S5**

**(A)** Representative CD117 staining of primary tumor tissues.

**(B)** Representative Ki67 staining of primary tumor tissues.

**(C)** TUNEL assay were used to determine the effects of HIF-1α and PGD expression alteration on cell apoptosis in the samples collected from nude mice. The error bars represent the mean (n = 3) ± S.D. *P < 0.05, **P < 0.01, ***P < 0.001.

**Supplementary**

**Table 1**

| hexokinase-Forward | GCTCTCCGATGAAACTCTCATAG |
| --- | --- |
| hexokinase-Reverse | GGACCTTACGAATGTTGGCAA |
| β-actin-Forward | CTCCATCCTGGCCTCGCTGT |
| β-actin-Reverse | GCTGTCACCTTCACCGTTCC |
| PFK-1-Forward | GGTGCCCGTGTCTTCTTTGT |
| PFK-1-Reverse | AAGCATCATCGAAACGCTCTC |
| isocitrate dehydrogenesa-Forward | TGTGGTAGAGATGCAAGGAGA |
| isocitrate dehydrogenesa-Reverse | TTGGTGACTTGGTCGTTGGTG |
| G6PD-Forward | CGAGGCCGTCACCAAGAAC |
| G6PD-Reverse | GTAGTGGTCGATGCGGTAGA |
| PGD-Forward | ATGGCCCAAGCTGACATCG |
| PGD-Reverse | AAAGCCGTGGTCATTCATGTT |
| Citrate synthase-Forward | TGCTTCCTCCACGAATTTGAAA |
| Citrate synthase-Reverse | CCACCATACATCATGTCCACAG |

**Table2**

|  | Primers |
| --- | --- |
| E-box-1 | Forward: 5'-TCCTCCTAGCAAGAAGGGACT-3'  Reverse :5'-CTATGTGACCCGCGAGATGG-3' |
| E-box-2 | Forward: 5'-CGGAGCGGAGGACACGAC-3'  Reverse :5'- TACAGTTTACCAGGCACTCGG -3' |
| E-box-3 | Forward: 5'- CCGGGGCAATGTCATCTAGG-3'  Reverse :5'- CATGAAGCCACACCATTGGC-3' |
