# Supplementary figures and images for: HIF-1α regulates cellular metabolism, and Imatinib resistance by targeting phosphogluconate dehydrogenase in gastrointestinal stromal tumors
Source: Cell Death Dis. 2020 Jul 27;11(7):586. doi: 10.1038/s41419-020-02768-4 (PMC7385157; doi:10.1038/s41419-020-02768-4)

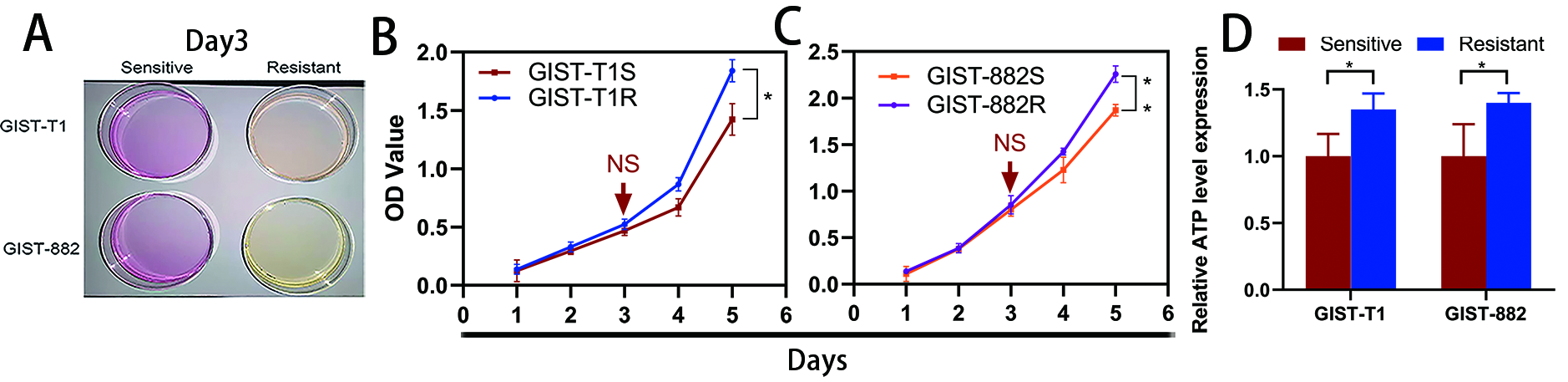

Supplement: Supplementary file 2 — Supplementary information 2 [file 41419_2020_2768_MOESM2_ESM.tif]

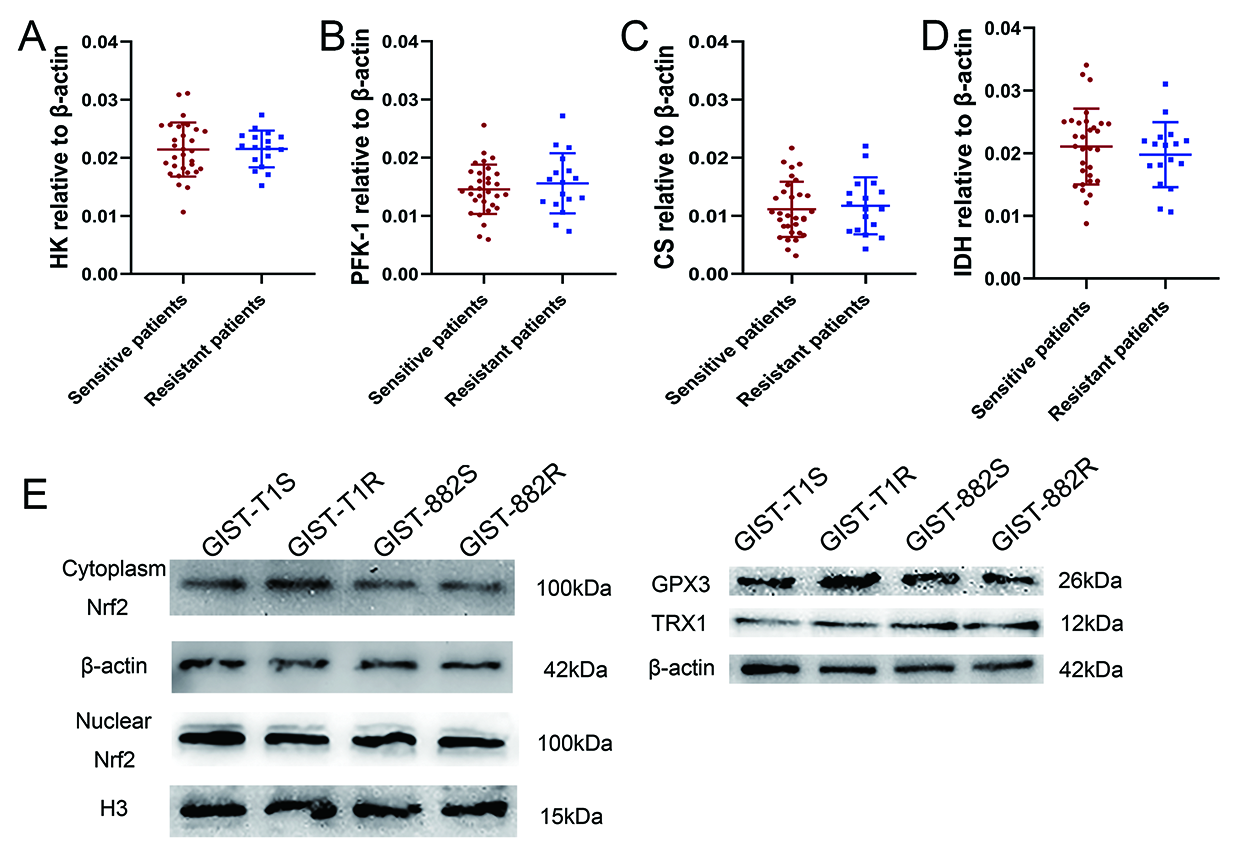

Supplement: Supplementary file 3 — Supplementary information 3 [file 41419_2020_2768_MOESM3_ESM.tif]

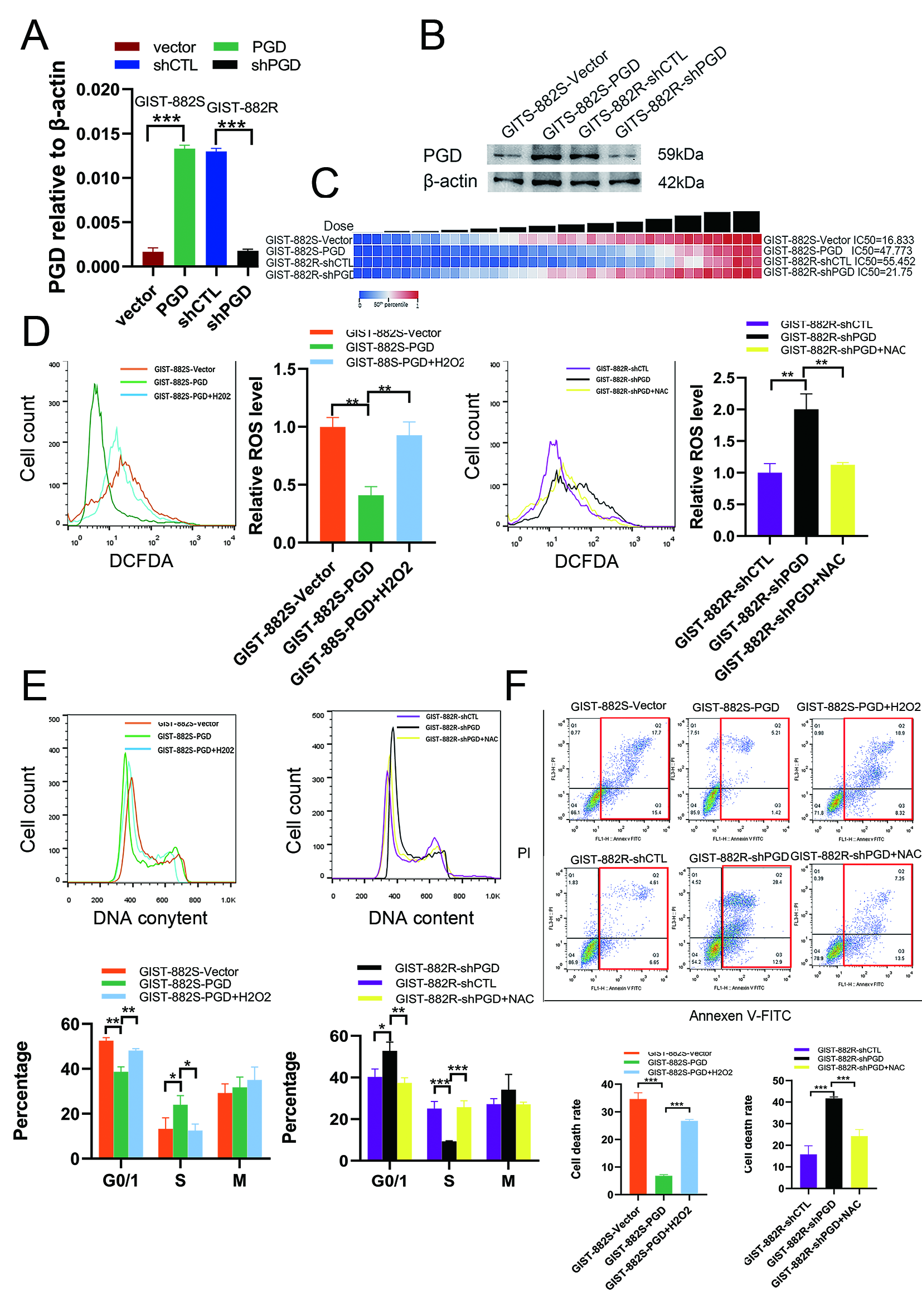

Supplement: Supplementary file 4 — Supplementary information 4 [file 41419_2020_2768_MOESM4_ESM.tif]

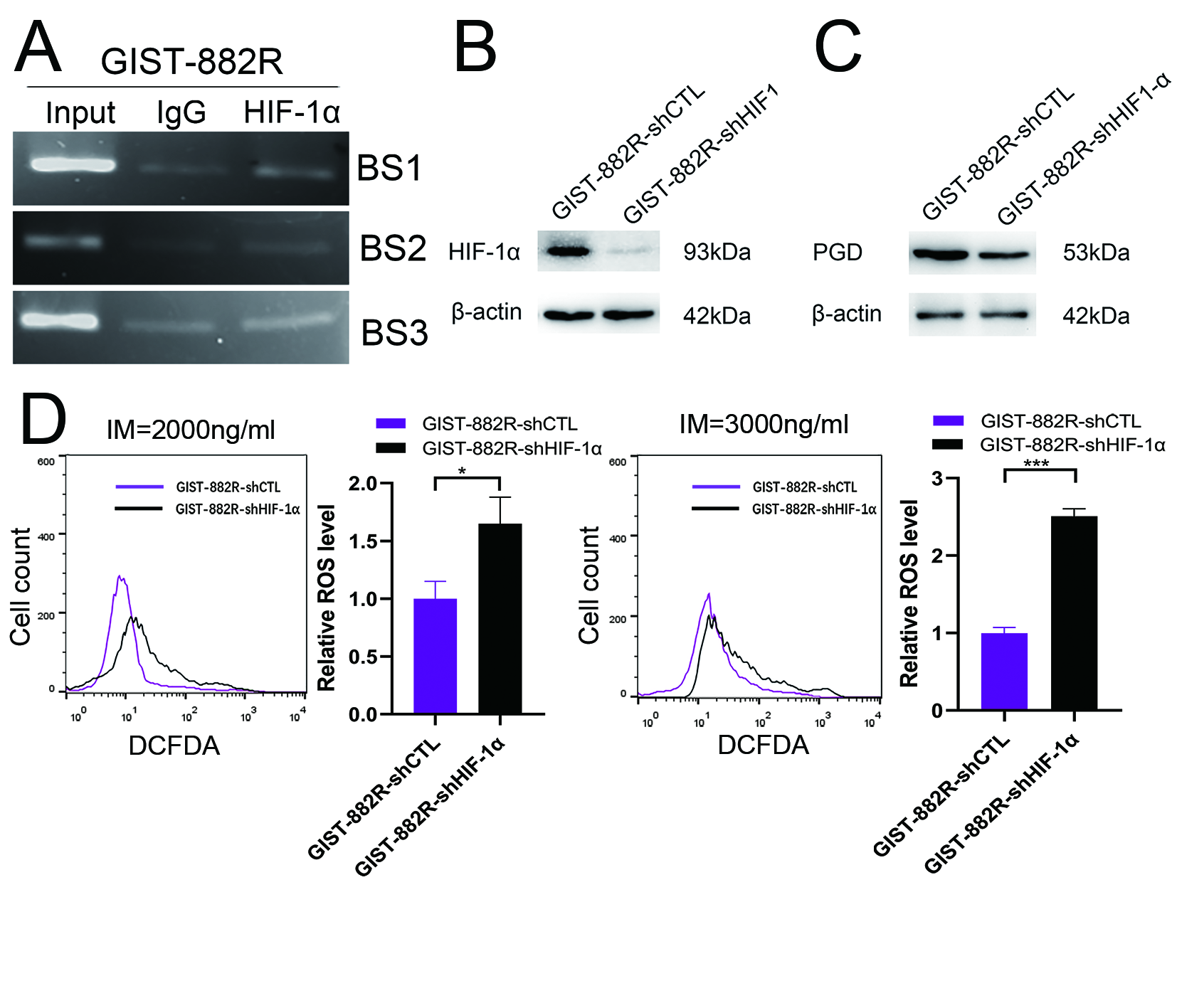

Supplement: Supplementary file 5 — Supplementary information 5 [file 41419_2020_2768_MOESM5_ESM.tif]

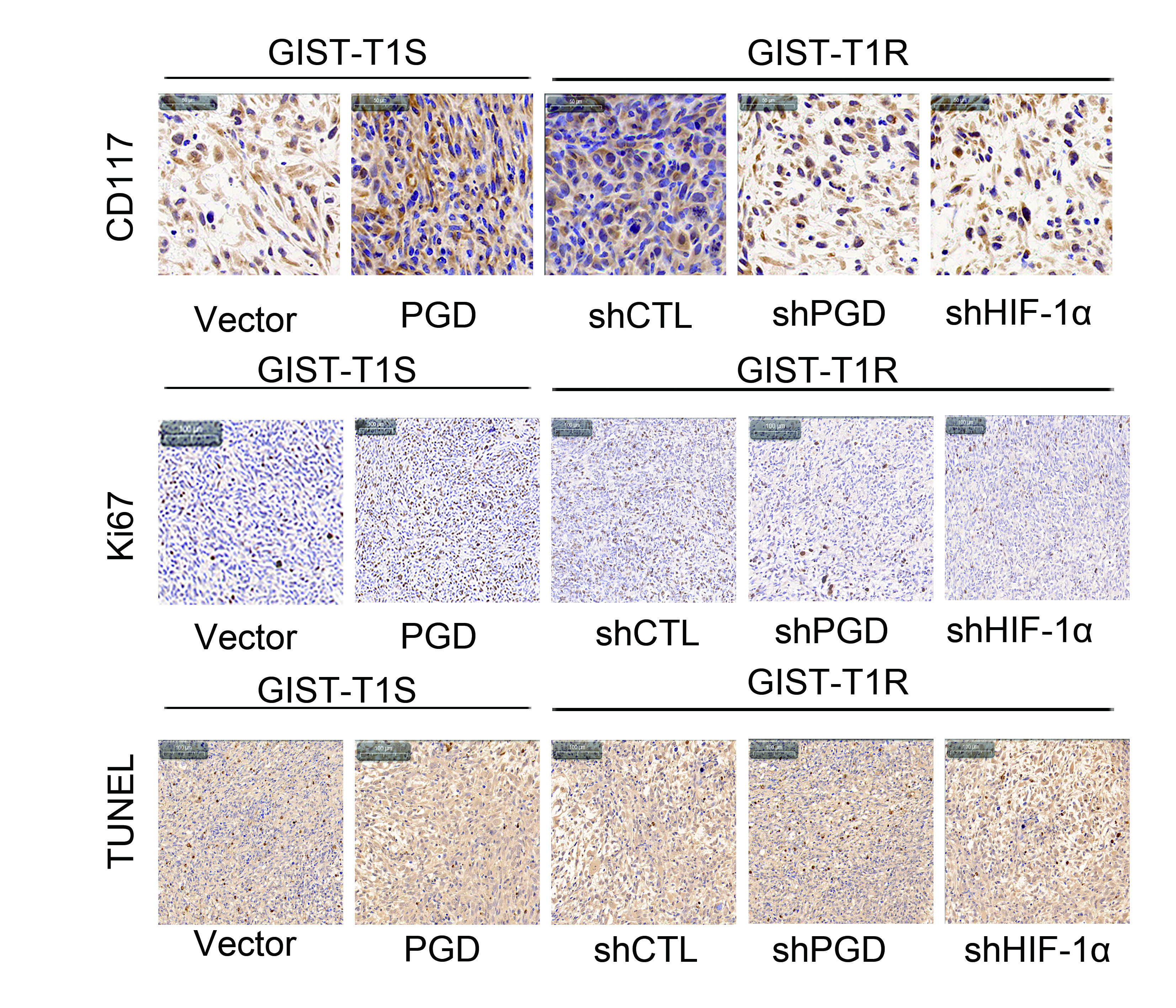

Supplement: Supplementary file 6 — Supplementary information 6 [file 41419_2020_2768_MOESM6_ESM.tif]
